# Psychometric properties of the personal financial wellness scale in Chilean young adults

**DOI:** 10.1186/s41155-026-00398-x

**Published:** 2026-06-11

**Authors:** Luis Mario Castellanos-Alvarenga, José Andrés Sepúlveda-Maldonado, Mauro P. Olivera, Jorge Schleef, Eduardo Sandoval-Obando

**Affiliations:** 1 Facultad de Ciencias Sociales y Educación, Escuela de Psicología, Universidad Santo Tomás, Temuco, Chile; 2 Departamento de Psicología, Universidad de La Frontera, Temuco, Chile; 3 Facultad de Psicología y Humanidades, Universidad San Sebastián, Puerto Montt, Chile; 4 Escuela de Psicología, Facultad de Ciencias Sociales y Humanidades, Universidad Autónoma de Chile, Temuco, Chile

**Keywords:** Financial well-beingfffund, Emerging adulthood, Structural equation modeling, Measurement invariance

## Abstract

**Background:**

Financial well-being is a key determinant of quality of life, since it reflects the individual's ability to meet current and ongoing financial obligations, feel secure about their financial future, and make choices that allow enjoyment of life. This is particularly important contexts with high inequality and uncertainty such as the case of Chile where many individuals rely on consumer credit from retail and banking institutions for monthly expenses. Therefore, having an instrument the reliably measures said variable across different sociodemographic groups, such as gender, ethnicity, and employment status is essential.

**Methods:**

This study examined the psychometric properties of the Personal Financial Wellness Scale (PFWS) among Chilean emerging adults. An adapted version of the PFWS was administered to 624 university and technical students (64.1% women), aged 18 to 29 years (M = 20.44, SD = 3.35). Exploratory Structural Equation Modeling (ESEM) was used to test the factorial structure, and multigroup analyses evaluated measurement invariance across gender, ethnicity, and employment status.

**Results:**

Results supported a two-factor model, financial distress and financial well-being, with satisfactory internal consistency and model fit. Partial measurement invariance was established across ethnicity and employment status, with changes in fit indices remaining within acceptable thresholds (ΔCFI ≤ .01, ΔRMSEA ≤ .015, ΔSRMR ≤ .030). For ethnicity, constraints on item 7 were released, and for employment status, intercepts of items 4 and 7 were relaxed. In contrast, full invariance was achieved across gender, with non-significant model comparisons (χ^2^(26) = 42.350, p = .023; χ^2^(52) = 61.946, *p* = .163) and stable fit indices across nested models (ΔCFI = 0.000, ΔRMSEA = 0.005, ΔSRMR = 0.013).

**Conclusions:**

These findings confirm that the PFWS captures essential dimensions of financial well-being in a culturally sensitive way, supporting its use as an early detection tool for financial distress and for guiding targeted interventions in socioeconomically vulnerable young adults.

## Introduction

Financial well-being is a key determinant of quality of life (Mathew et al., 2022), as it reflects an individual's ability to meet current and ongoing financial obligations, feel secure about their financial future, and make choices that allow enjoyment of life (Tahir & Ahmed, 2021). Beyond economic sufficiency, it encompasses perceptions of control (Bialowolski et al., 2021a), autonomy (Kumar et al., 2023), and mental health regarding financial matters (Ryu & Fan, 2023), influencing relational stability (Kelley et al., 2023), and overall life satisfaction (Foong et al., 2021).

This multidimensional view of financial well-being becomes particularly relevant in contexts where high levels of debt and financial insecurity are prevalent (Simonse et al., 2024).

In Chile, over 57% of households are in debt and many individuals rely on consumer credit from retail and banking institutions (Banco Central Chile, 2021). Moreover, 30.1% of young adults between 18 and 29 years report being heavily indebted, a figure that increases among women and younger adults (Instituto Nacional de la Juventud [INJUV], 2020). The growing acceptance of credit use and consumer debt, particularly among emerging adults, has generated increasing concern about their susceptibility to financial stress and psychological vulnerability (Castellanos-Alvarenga & Denegri-Coria, 2024). While financial stress has traditionally been associated with external conditions like income instability or inflation (Friedline et al., 2021), it also reflects subjective perceptions of financial inadequacy and insecurity (Kasal, 2023).

The consequences of poor financial well-being include increased financial stress (Tran et al., 2025), reduced academic and job performance (Rosso et al., 2024), and lower subjective well-being (Rahman et al., 2021). Poor personal financial wellness has been consistently associated with adverse psychological outcomes, including higher levels of stress, anxiety, and depressive symptoms (Bialowolski et al., 2021a, 2021b; Guan et al., 2022). It may also impair sleep quality (Du et al., 2021), increases substance use (Nigatu et al., 2024), and negatively affects the quality of interpersonal relationships (Peetz et al., 2024). These associations are particularly relevant during emerging adulthood, a stage characterized by greater economic instability, increased vulnerability to mental health problems, and the pursuit of financial independence(Barrera-Herrera et al., 2019). This life period, situated between adolescence and full adulthood, involves significant developmental transitions that often intensify financial pressures and emotional demands (Felinto et al., 2020; Meier, 2020).

Therefore, accurate assessment of personal financial wellness in young adults is not only important for financial behavior research, but also for identifying individuals at risk of psychological comorbidities and for informing early preventive strategies. To address this, several studies have highlighted the need for accurate and context-sensitive instruments to assess personal financial well-being in emerging adults (Castellanos-Alvarenga et al., 2022; She et al., 2023; Vosylis & Klimstra, 2020).

The Personal Financial Wellness Scale (PFWS) is one of the most widely used instruments to measure financial well-being as a multidimensional construct, incorporating both objective and subjective aspects (Sabri & Aw, 2020). More recently, Buabang et al. (2022) evaluated the PFWS in Germany, Italy, Netherlands, Slovenia, Spain, the United Kingdom, and the United States, finding excellent reliability and evidence of convergent and discriminant validity. However, their results also showed mixed evidence for the expected one-factor structure, with better fit for a modified one-factor model, and metric invariance only across Slovenia, Spain, the United Kingdom, and the United States, while scalar invariance was not supported. This suggests caution when using the scale for direct cross-country comparisons.

Additional validation and adaptation studies have extended the use of the instrument to other contexts. In Brazil, De Carvalho et al. (2021) translated and culturally adapted the IFDFW into Brazilian Portuguese, reporting strong test–retest reliability and good internal consistency. In Peru, Millones-Liza and García-Salirrosas (2024) provided evidence of validity and reliability for the IFDFW in citizens from the coast, Andes, and rainforest regions, supporting an eight-item one-dimensional model with correlated errors and adequate reliability. In South Korea, Kim et al. (2025) adapted the Korean version of the PFWS in a large adult sample and found support for a single-factor structure, satisfactory internal consistency, and evidence of measurement invariance across gender and regions. Similarly, recent adaptations have been developed in Arabic-speaking Lebanese adults and in Chinese lung cancer patients and caregivers, further supporting the cross-cultural applicability of the scale in diverse populations (El Zouki et al., 2025; Jia et al., 2025). Overall, these studies suggest that the PFWS is a brief and reliable measure of subjective financial well-being, although its dimensionality and measurement invariance may vary across cultural contexts. Therefore, additional evidence in Latin American youth remains necessary, particularly in Chile, where structural inequalities intersect with financial instability (Araujo, 2019), which is reflected in high levels of student debt (Sepúlveda-Maldonado et al., 2022), and precarious job conditions (Comisión para el Mercado Financiero [CMF], 2025; Marambio-Tapia, 2021). These socioeconomic dynamics underscore the need for a rigorous evaluation of the scale’s validity and measurement properties within the Chilean context.

Beyond establishing internal consistency and factorial structure, it is essential to test the measurement invariance of the PFWS across key sociodemographic groups, particularly gender, ethnicity, and employment status., This issue is especially relevant in the Region of La Araucanía, where approximately 32.82% of the population self-identifies as Mapuche (Instituto Nacional de Estadística, 2018). The persistence of structural and cultural inequalities affecting Mapuche communities (Castillo et al., 2022; Delgado et al., 2025) raises the need to determine whether the PFWS functions equivalently across ethnic groups. Likewise, gender and employment status are key variables in the Chilean socio-economic landscape, as women and unemployed youth are disproportionately affected by financial vulnerability (Baquedano-Rodríguez et al., 2025; Organisation for Economic Co-operation and Development [OECD], 2022).

Without establishing invariance, group comparisons may yield misleading conclusions, undermining both the interpretability of research findings and the equity of interventions based on such data. Addressing this gap, the present study aims to examine the factorial validity of the Personal Financial Wellness Scale (PFWS) and to evaluate its measurement invariance across gender, ethnicity, and employment status in a sample of emerging adults from the Region of La Araucanía, Chile.

## Methodology

### Participants

The study utilized a cross-sectional design with a correlational and explanatory scope, applying a non-probabilistic purposive sampling strategy. Data were gathered through an online questionnaire administered to higher education students in the Araucanía Region, Chile, enabling the assessment of the factorial structure, internal consistency, and measurement invariance of the Personal Financial Wellness Scale across gender, ethnicity, and employment status at a single point in time.

The study was conducted using a cross-sectional design with a correlational and explanatory approach, based on a non-probabilistic purposive sampling method (Creswell, 2014). The sample size was calculated a priori to ensure statistical power and reliable parameter estimates for structural equation modeling analyses, with Exploratory Structural Equation Modeling (ESEM) approach (Soper, 2024). ESEM is an advanced statistical technique that integrates features of exploratory factor analysis within a structural equation modeling framework, allowing for more flexible modeling of latent constructs by permitting cross-loadings (Morin, 2023).

The final sample consisted of 624 higher education students, of whom 63.1% identified as female. The average age was 20.44 years (SD = 3.35). Inclusion criteria were: (1) being between 18 and 29 years old, (2) being enrolled in a technical or professional degree program between the first and fifth year, and (3) studying at an educational institution located in the Araucanía Region in southern Chile. Table 1 presents the sociodemographic characteristics of the sample.

**Table 1:** Sociodemographic characteristics of the sample, separated by gender

Characteristic	Male		Female		Total		Group comparison
	*n*	%	*n*	%	*n*	%	
Participants	224	35.9	400	64.1	624	100	
Age (*M*; *SD*)	20.81 (3.19)		20.23 (3.44)		20.44 (3.36)		*t*(491.01) = −2.14, *p* =.033
Socioeconomic status							
Low	5	2.3	23	5.9	28	4.6	*χ*^*2*^(2) = 4.18, *p* =.124
Middle	190	87.6	326	83.6	516	85.0	
High	22	10.1	41	10.5	63	10.4	
Ethnicity							
Does not identify	169	75.4	307	76.8	476	76.3	*χ*^*2*^(1) = 0.14, *p* =.713
It identifies	55	24.6	93	23.3	148	23.7	
Type of institution attended							
University	124	62.0	301	88.8	425	78.8	*χ*^*2*^(2) = 58.55, *p* <.001
Technical Center	50	25.0	17	5.0	67	12.4	
Professional Institute	26	13.0	21	6.2	47	8.7	
Employment situation							
It is not employed	143	63.8	277	69.3	420	67.3	*χ*^*2*^(1) = 1.91, *p* =.167
It is employed	81	36.2	123	30.8	204	32.7	
Credit card							
It does not use	176	78.6	330	82.5	506	81.1	*χ*^*2*^(1) = 1.45, *p* =.229
It uses	48	21.4	70	17.5	118	18.9	
Income (*M*; *SD*)*	1.34 (1.39)		1.43 (1.62)		1.40 (1.54)		*t*(526) = 0.67, *p* =.504
Debt (*M*; *SD*)*	0.11 (0.50)		0.18 (1.17)		0.16 (0.98)		*t*(622) = 0.91, *p* =.362

*N* absolute frequency, *%* percentage, *M* mean, *SD* standard deviation

*p* = significance level

^*^Income and debt per each 100,000 Chilean pesos (CLP)

### Measuring instruments

To assess financial well-being, the Personal Financial Wellness Scale (Prawitz et al., 2006) was used. For this study, the adaptation began from the Spanish version employed by (Lobos et al., 2021) in a sample of Ecuadorian health workers. This version served as the basis for a cultural and linguistic adaptation to the Chilean context, which included modifications to vocabulary to ensure compatibility with Chilean Spanish and adjustments to reflect the local currency, as recommended in instrument adaptation guidelines (Cruchinho et al., 2024; Fenn et al., 2020).

In addition, after obtaining permission to adapt the instrument, the original 10-point response format was shortened to a 6-point scale ranging from 1 (*not at all financially healthy*) to 6 (*completely financially healthy*). This decision was based on methodological evidence suggesting that increasing the number of response categories does not necessarily improve measurement quality and may increase cognitive burden when respondents are required to distinguish between highly similar adjacent categories. Previous research has shown that scales with 4, 5, or 6 response categories tend to show comparable psychometric performance, whereas very short response formats may reduce reliability and discriminating power (Lee & Paek, 2014). Similarly, evidence on response scale design indicates that scales with fewer, clearly interpretable categories may yield better data quality than longer formats with excessive response options (Revilla et al., 2013). Likewise, simulation-based evidence suggests that reliability and validity are generally optimized when response formats include between four and seven categories (Lozano et al., 2008). Content validity was established through the review of three academic experts in financial behavior and psychometrics, who independently evaluated the relevance, clarity, and cultural adequacy of the items.

The expert judgments demonstrated high agreement. Following this, ten cognitive interviews were conducted with individuals from the target population, including five participants who self-identified as Mapuche and five as non-Mapuche, to assess comprehension and semantic clarity of the items. Based on their feedback, minor adjustments were made to improve wording. The final instrument retained the original eight items, which assess perceived financial control, security, and stability. The overall score was calculated by averaging the item responses.

Previous research has consistently supported a unidimensional factor structure of the PFWS, with high internal consistency (α >.85) reported across studies (Mahdzan et al., 2019). Table 2 presents the means and standard deviations found in the current sample as well as the structure found in the present study.

**Table 2:** Descriptive Statistics of the Personal Financial Wellness Scale

Items	*M*	*SD*
1. What do you think is your current level of financial stress today?	3.679	1.555
4. How often do you worry about being able to cover normal monthly living expenses?	4.048	1.557
6. How often would you like to go out to eat, go to the movies, or do something else and you don’t because you can’t afford it?	3.726	1.623
7. How often do you manage on your own financially to get through the day?	3.196	1.581
8. How stressed do you feel about your personal finances in general?	3.752	1.677
2. How satisfied are you with your current financial situation?	2.981	1.423
3. How do you feel about your current financial situation?	3.095	1.420
5. How confident are you that you could find the money to pay for a financial emergency costing around $800,000 CLP?	2.377	1.575

All items use a six-point response scale, *M* Mean, *SD* Standard Deviation

### Procedure

The research protocol was reviewed and approved by a university-based Research Ethics Committee in Chile, in accordance with the principles outlined in the Declaration of Helsinki. Participants were invited to complete the study instruments via an online survey platform. The estimated response time was approximately 15 min. All participants provided digital informed consent prior to beginning the questionnaire. Participation was entirely voluntary, and no financial or material incentives were offered. Anonymity and confidentiality were assured throughout the data collection process.

### Statistical analyses

Data base was downloaded from the QuestionPro platform and processed using RStudio version 2023.12.0 + 369 (Posit team, 2025). Primary analyses were conducted with the esemComp package (Silvestrin & de Beer, 2022).

Prior to analysis, multivariate outliers were excluded based on Mahalanobis distance, and descriptive analyses were performed to characterize the sample. To examine the factorial structure of the PFWS, three theoretically plausible alternative models were compared: (a) a one-factor CFA model, representing financial well-being as a general construct; (b) a two-factor CFA model, specifying financial stress and financial well-being as correlated but distinct dimensions; and (c) a two-factor ESEM model, allowing cross-loadings to be estimated among conceptually related indicators. This strategy was selected because the PFWS items assess closely related subjective financial experiences. In this regard, fixing cross-loadings to zero a priori, as in conventional CFA, could impose an overly restrictive representation of the construct. Therefore, the ESEM approach was used as a flexible confirmatory strategy (Morin, 2023) and was estimated using the *ESEM-within-CFA* approach (Marsh et al., 2020) applying *GeominQ* rotation and the Weighted Least Squares Mean and Variance Adjusted Estimator (WLSMV), which is recommended for ordinal data (DiStefano & Morgan, 2014; Park, 2023). Model fit was evaluated using the following criteria: (a) Comparative Fit Index (CFI) and Tucker-Lewis Index (TLI), with values ≥.90 indicating acceptable fit and ≥.95 indicating excellent fit; (b) Root Mean Square Error of Approximation (RMSEA) and Standardized Root Mean Square Residual (SRMR), with values ≤ 0.08 indicating acceptable fit and ≤.06 indicating excellent fit (Marsh et al., 2005; Shi et al., 2019).

The following models were compared to assess the factorial structure of the scale, and the best-fitting model was selected based on recommendations from the ESEM literature (Morin, 2023; Morin et al., 2016): (a) a unidimensional model to test for irrelevant multidimensionality; (b) the original two-factor model estimated using a confirmatory factor analysis (CFA) approach; and (c) a two-factor ESEM model to evaluate the potential presence of meaningful cross-loadings.

Subsequently, a multigroup analysis was conducted based on the selected factorial solution to test for measurement invariance across gender, ethnicity, and employment situation. A sequence of increasingly restrictive models was estimated: (a) configural invariance, allowing item loadings to vary freely across groups but retaining the same factor structure; (b) metric invariance, constraining factor loadings to be equal across groups; (c) scalar invariance, constraining item intercepts; and (d) residual invariance, constraining residual variances (Widaman & Helm, 2023). Model fit at each level of invariance was compared to the configural model.

Where full invariance was not achieved, equality constraints were relaxed based on the Wald test (Kline, 2023). Model comparisons were evaluated using changes in fit indices, with the following thresholds used to detect non-invariance (ΔCFI and ΔTLI <.010, ΔRMSEA y ΔSRMR <.015; Chen, 2007; Morin et al., 2016). Finally, internal consistency reliability was assessed using McDonald’s Omega coefficient with a 95% confidence interval (Hayes & Coutts, 2020), computed via the *semTools* package (Jorgensen et al., 2022).

## Results

Table 2 presents the descriptive statistics for the items of the *Personal Financial Wellness Scale* in the study sample. Overall, the item means suggest moderate levels of perceived financial strain and financial well-being, with higher scores observed in items related to worry about covering monthly living expenses and general financial stress. In contrast, the lowest mean was found for the item assessing confidence in being able to cover an unexpected financial emergency, suggesting that perceived capacity to respond to unforeseen expenses may represent a particularly vulnerable aspect of participants’ financial well-being.

Regarding the structure of the scale, the two-factor correlated model estimated using the ESEM approach (Model 1) showed excellent fit indices and well-defined factors based on their primary loadings (λFactor 1 = 0.464 to 0.823; λFactor 2 = 0.486 to 0.887), with weak cross-loadings (|λFactor 1|= 0.025 to 0.027; |λFactor 2|= 0.069 to 0.190) and low covariances between the factors (r = −0.239; *p* < 0.001). On the other hand, the one-factor model (Model 2) did not show acceptable fit, which supports the rejection of the unidimensionality of the construct. Additionally, although the two-factor model estimated using the CFA approach (Model 3) showed acceptable fit indices, its overall fit was substantially poorer compared to Model 1 (ΔCFI = −0.029; ΔTLI = −0.046; ΔRMSEA = + 0.038). Therefore, the ESEM solution appears to be optimal, as it allows for the presence of cross-loadings (see Tables 3 and 4). This factorial structure showed adequate internal consistency indices for both factors (ωdistress = 0.759, CI 95% [0.725—0.793]; ωwell-being = 0.795, CI 95% [0.765—0.825]).

**Table 3:** Goodness-of-fit indices for alternative models of the factorial structure

Model	χ^2^(*df*)	CFI	TLI	RMSEA (IC 90%)	SRMR	ΔCFI	ΔTLI	ΔRMSEA	ΔSRMR
M1	32.455 (13)	1.000	1.004	0.028 (0.016–0.041)	0.024	-	-	-	-
M2	343.558 (20)	0.732	0.625	0.180 (0.163–0.197)	0.149	−0.268	−0.379	+ 0.152	+ 0.125
M3	100.220 (19)	0.971	0.958	0.066 (0.054–0.079)	0.058	−0.029	−0.046	+ 0.038	+ 0.034

*M1* Two-factor model with ESEM approach, *M2* One-factor model with CFA approach, *M3* Two-factor model with CFA approach, *Δ* change in goodness-of-fit index

**Table 4:** Factor loadings for the estimated models

Items	M1			M3		
	λ_distress_	λ_well-being_	δ	λ_distress_	λ_well-being_	δ
Item 1	**0.653**	−0.069	0.547	0.689		0.526
Item 2	−0.025	**0.887**	0.202		0.898	0.193
Item 3	−0.027	**0.854**	0.258		0.868	0.247
Item 4	**0.621**	0.190	0.635	0.498		0.752
Item 5	−0.027	**0.486**	0.756		0.486	0.764
Item 6	**0.464**	−0.130	0.739	0.527		0.723
Item 7	**0.531**	0.128	0.735	0.452		0.796
Item 8	**0.823**	−0.081	0.285	0.883		0.220

*λ* standardized factor loadings, *δ* residual variances

Measurement invariance models by gender, ethnicity, and employment status were subsequently estimated, all based on the ESEM solution (see Table 5).

**Table 5:** Goodness-of-fit indices for measurement invariance models by gender, ethnicity, and employment status

Model	χ^2^(*df*)	*p*	CFI	TLI	RMSEA (90% IC)	SRMR	ΔCFI	ΔTLI	ΔRMSEA	ΔSRMR
Measurement invariance by gender										
Configural	42.350 (26)	0.023	1.000	1.019	0.026 (0.010–0.040)	0.024	-	-	-	-
Metric	46.237 (38)	0.169	1.000	1.015	0.021 (0.000–0.040)	0.032	0.000	−0.004	−0.005	+ 0.008
Scalar	53.631 (44)	0.152	1.000	1.014	0.021 (0.000–0.039)	0.034	0.000	−0.005	0.005	+ 0.010
Residual	61.946 (52)	0.163	1.000	1.013	0.021 (0.000–0.038)	0.037	0.000	−0.006	0.005	+ 0.013
Measurement invariance by ethnic										
Configural	39.829 (26)	0.041	1.000	1.020	0.024 (0.005–0.038)	0.023	-	-	-	-
Metric	53.303 (38)	0.051	1.000	1.009	0.030 (0.000–0.047)	0.035	0.000	−0.011	+ 0.006	+ 0.012
Partial metric *	42.489 (37)	0.246	1.000	1.016	0.018 (0.000–0.038)	0.030	0.000	−0.004	−0.006	+ 0.007
Measurement invariance by employment status										
Configural	37.369 (26)	0.069	1.000	1.020	0.023 (0.000–0.039)	0.023	-	-	-	-
Metric	36.242 (38)	0.551	1.000	1.020	0.000 (0.000–0.031)	0.027	0.000	0.000	−0.023	+ 0.004
Scalar	75.623 (44)	0.002	0.999	0.999	0.041 (0.024–0.056)	0.040	−0.001	−0.021	+ 0.018	+ 0.017
Partial metric **	51.744 (42)	0.144	1.000	1.012	0.023 (0.000–0.042)	0.033	0.000	−0.008	0.000	+ 0.010

^*^ = Equality constraints on the factor loading of item 7 were released

^**^ = Equality constraints on the intercepts of items 4 and 7 were released

The analysis began with the configural invariance model, used as the baseline for comparison. In this model, no equality constraints are imposed on the parameters, aside from maintaining the same measurement structure across groups. In the case of gender invariance, the metric, scalar, and residual models all demonstrated acceptable fit indices and did not show substantial deterioration in comparison to the configural model. These results indicate that the items function equivalently across men and women at the configural, metric, scalar, and residual levels. Therefore, the PFWS provides a measurement basis that would support latent mean comparisons by gender in future analyses.

For the ethnicity invariance analysis, although the metric invariance model exhibited excellent fit indices, its overall fit deteriorated substantially compared to the configural model. The Lagrange Multiplier test indicated that releasing item 7 would significantly improve model fit. Consequently, a partial metric invariance model was estimated by removing the equality constraint on item 7. This adjusted model showed a considerable improvement in fit indices, with no substantial differences from the configural model. This result suggests that most items contributed similarly to the latent factors across groups, although Item 7 showed differential functioning in its factor loading. Therefore, the scale may be used with caution to compare associations between financial well-being and other variables across ethnic groups. However, because scalar invariance was not established, latent mean comparisons by ethnicity are not recommended.

Finally, regarding measurement invariance by employment status, the metric invariance model demonstrated excellent fit and did not differ substantially from the configural model, indicating that all items are interpreted similarly by individuals with and without paid employment. However, although the scalar invariance model also showed excellent fit indices, its overall fit deteriorated considerably relative to the configural model. Table 6 shows the factor loadings for the invariance models by gender, ethnicity, and employment status.

**Table 6:** Factor loadings for the invariance models by gender, ethnicity, and employment status

Item	Gender							
	Male				Female			
	λ_distress_	λ_well-being_	τᵢ	δᵢ	λ_distress_	λ_well-being_	τᵢ	δᵢ
1	0.645	−0.069	2.318	0.562	0.657	−0.072	2.266	0.538
2	−0.026	0.865	2.161	0.243	−0.026	0.877	2.049	0.219
3	−0.023	0.864	2.259	0.245	−0.023	0.876	2.143	0.221
4	0.612	0.187	2.532	0.634	0.634	0.199	2.518	0.627
5	−0.021	0.473	1.528	0.772	−0.022	0.498	1.502	0.746
6	0.454	−0.128	2.260	0.756	0.466	−0.134	2.224	0.731
7	0.522	0.124	1.957	0.737	0.541	0.132	1.946	0.729
8	0.811	−0.083	2.179	0.311	0.816	−0.086	2.105	0.290
Item	Ethnicity							
	Does not identify				It identifies			
	λ_distress_	λ_well-being_	τᵢ	δᵢ	λ_distress_	λ_well-being_	τᵢ	δᵢ
1	0.677	−0.056	2.304	0.520	0.599	−0.063	2.598	0.622
2	−0.025	0.866	2.132	0.239	−0.021	0.926	1.985	0.135
3	−0.027	0.834	2.198	0.292	−0.024	0.919	2.129	0.146
4	0.639	0.192	2.539	0.617	0.578	0.221	2.813	0.670
5	−0.025	0.487	1.509	0.756	−0.021	0.515	1.505	0.729
6	0.470	−0.127	2.247	0.733	0.405	−0.140	2.473	0.793
7	0.493	0.130	2.001	0.773	0.677	0.137	2.093	0.561
8	0.845	−0.081	2.190	0.245	0.717	−0.088	2.412	0.452
Item	Employment situation							
	Not employed				Employed			
	λ_distress_	λ_well-being_	τᵢ	δᵢ	λ_distress_	λ_well-being_	τᵢ	δᵢ
1	0.671	−0.068	2.304	0.527	0.617	−0.061	2.350	0.592
2	−0.024	0.889	2.014	0.201	−0.025	0.895	2.298	0.184
3	−0.028	0.877	2.161	0.220	−0.026	0.800	2.232	0.346
4	0.592	0.187	2.426	0.659	0.596	0.185	2.995	0.681
5	−0.047	0.511	1.535	0.727	−0.041	0.435	1.483	0.797
6	0.491	−0.128	2.256	0.717	0.438	−0.111	2.233	0.764
7	0.528	0.126	1.929	0.732	0.463	0.108	2.224	0.806
8	0.830	−0.080	2.122	0.278	0.807	−0.076	2.288	0.304

λ = standardized factor loading

τ = item intercept

δ = residual variance

The Lagrange Multiplier test suggested releasing the intercept constraints for items 4 and 7, and the model was subsequently re-estimated with these parameters freed. This partial scalar invariance model maintained acceptable fit indices without substantial differences from the configural model. These findings indicate that future latent mean comparisons by employment status should account for these non-invariant intercepts.

## Discussion

This study provides empirical support for the psychometric validity of the Personal Financial Wellness Scale (PFWS) among Chilean emerging adults, in line with the growing recognition of financial well-being as a central component of quality of life (Mathew et al., 2022). The findings reinforce the notion that personal financial wellness is a multidimensional construct encompassing not only economic sufficiency but also individuals’ perceived ability to meet obligations, feel secure about the future, and make choices aligned with their life goals (Tahir & Ahmed, 2021).

The scale demonstrated a two-factor structure (financial distress and financial well-being) that reflects the emotional and cognitive dimensions associated with managing personal finances. While the original purpose of authors such as Mathew et al. (2022) was not to address measurement models, their conceptualization supports the importance of assessing both negative and positive financial experiences. This structure is especially pertinent in the Chilean context, where more than half of households are in debt and financial insecurity is pervasive (Banco Central de Chile, 2021), particularly among young people (INJUV, 2020). The internal consistency indices and model fit support the PFWS as a reliable tool to capture these financial realities. The application of Exploratory Structural Equation Modeling (ESEM) offered a more flexible representation of the scale’s factorial structure, enabling modest cross-loadings that may reflect overlapping perceptions of control, sacrifice, and satisfaction, components that are central to subjective financial well-being (Bialowolski et al., 2021b; Kumar et al., 2023). Although the two-factor CFA model also showed acceptable fit and may represent a viable and more parsimonious alternative, the two-factor ESEM solution was retained because it provided a less restrictive and theoretically more realistic representation of the PFWS. This model allowed cross-loadings to be estimated while preserving a clear two-factor structure, with well-defined primary loadings and interpretable dimensions of financial stress and financial well-being. Nevertheless, the adequate fit of the CFA model suggests that future studies could consider both solutions, depending on whether the analytical priority is parsimony or a more flexible representation of item complexity.

Consistent with findings from national reports and recent studies (Castellanos-Alvarenga & Denegri-Coria, 2024; Sepúlveda-Maldonado et al., 2022), descriptive analyses showed higher financial distress among participants, suggesting that debt and economic uncertainty are internalized as emotional discomfort, insecurity, and limited autonomy. These psychological consequences, including stress, anxiety, and depressive symptoms (Guan et al., 2022; Ryu & Fan, 2023), underscore the importance of financial wellness not only as an economic indicator but as a relevant factor in mental health promotion during emerging adulthood (Barrera-Herrera et al., 2019; Meier, 2020).

The invariance findings should be interpreted according to the specific level of equivalence achieved for each grouping variable. For gender, full scalar and residual invariance indicate that the PFWS measures financial stress and financial well-being equivalently across men and women, thereby supporting latent mean comparisons. For ethnicity, only partial metric invariance was achieved, suggesting that the scale may be used to examine associations between financial well-being and external variables across groups, but not to compare latent means. For employment status, partial scalar invariance indicates that latent mean comparisons are possible only after accounting for the non-invariant intercepts of Items 4 and 7. These results reinforce the need to avoid unadjusted comparisons based on observed sum scores, particularly when examining ethnicity or employment status. In particular, the need to relax constraints on items related to daily management and sacrifice may reflect different lived financial experiences among subgroups, especially among women, unemployed youth, and Mapuche participants, who are disproportionately affected by systemic inequities (Baquedano-Rodríguez et al., 2025; Castillo et al., 2022; Delgado et al., 2025).

The PFWS’s acceptable performance across these sociodemographic groups is especially relevant in La Araucanía, a region with a high proportion of Indigenous population and persistent structural and cultural inequalities. The ability to assess financial wellness fairly across such groups is essential to advancing equitable and context-sensitive interventions, as recommended by prior research (Araujo, 2019; OECD, 2022).

Taken together, the findings suggest that the PFWS is a psychometrically sound instrument for evaluating personal financial wellness in emerging adults in Chile. Its use can contribute not only to understanding economic behavior but also to the early identification of psychological risk factors associated with financial distress. Given the increasing visibility of student debt, informal credit, and labor precariousness in Chile (Marambio-Tapia, 2021; CMF, 2025), systematic monitoring of financial wellness may inform targeted educational and policy strategies.

The findings of this study should be interpreted in light of some methodological limitations. First, the use of a non-probabilistic sampling strategy limits the generalizability of the results to the broader population of Chilean young adults. Although the sample provided relevant evidence regarding the psychometric performance of the PFWS in this context, future studies should consider probability-based or more diverse sampling strategies to strengthen external validity. Second, the cross-sectional design prevents conclusions about temporal stability, causal relationships, or changes in financial well-being over time. Therefore, the present results should be understood as evidence of the scale’s functioning at a specific point in time rather than as an assessment of longitudinal invariance or developmental change.

Future research should include longitudinal designs to assess changes over time and examine how contextual factors such as inflation, educational costs, and employment instability influence financial wellness. Additionally, predictive analyses may help determine the role of financial well-being in academic performance, mental health outcomes, and interpersonal functioning, further validating the PFWS in diverse real-world contexts.

## Data Availability

The dataset will be made available upon request via email to the corresponding author.
